# High-Sensitivity C-Reactive Protein and Cancer

**DOI:** 10.2188/jea.JE20100128

**Published:** 2011-05-05

**Authors:** Seounghee Lee, Jae-Won Choe, Hong-Kyu Kim, Joohon Sung

**Affiliations:** 1 Health Promotion Center, Asan Medical Center, University of Ulsan College of Medicine, Seoul, South Korea; 2 Department of Epidemiology, School of Public Health and Institute of Health and Environment, Seoul National University, Seoul, South Korea

**Keywords:** high-sensitivity C-reactive protein, cancer, inflammation

## Abstract

**Background:**

High-sensitivity C-reactive protein (hs-CRP) is a commonly used inflammatory marker. The association between hs-CRP and cancer is less consistent than that between hs-CRP and cardiovascular diseases. This study explored the association between hs-CRP and cancer, using a large database of Korean health examination records.

**Methods:**

A total of 80 781 Koreans who visited the health promotion center of a general hospital were included. There were 729 cases of cancer of any primary site during a 3-year period. Subjects with a known cancer or a condition capable of affecting hs-CRP were excluded.

**Results:**

Serum hs-CRP was significantly higher in cancer cases (2.9 mg/L) than in non-cases (1.4 mg/L; *P* < 0.0001). With the lowest hs-CRP category (<1 mg/L) as reference, the crude odds ratios (ORs) for cancer were 1.36 (95% confidence interval [CI] = 1.16–1.62) for the second highest category (1–3 mg/L) and 2.49 (95% CI = 2.02–3.07) for the highest category (>3 mg/L), and the adjusted ORs for cancer were 1.16 (95% CI = 0.95–1.42) for the second highest category and 1.94 (95% CI = 1.51–2.51) for the highest category. After excluding cancer cases detected within 1 year after the check-up, the associations remained, although the reduced number of cancer cases (*n* = 88) attenuated the significance of the associations.

**Conclusions:**

Serum hs-CRP was positively associated with the risk of cancer, although causality cannot be inferred in this cross-sectional study. The results support the hypothesis that chronic inflammation plays a role in cancer.

## INTRODUCTION

High-sensitivity C-reactive protein (hs-CRP), an acute-phase plasma protein that increases during systemic inflammation, is one of the most frequently used inflammatory markers. CRP is produced primarily in the liver and is regulated by proinflammatory cytokines, especially interleukin-6. CRP levels in blood are normally very low and difficult to detect in healthy individuals, but increase rapidly with inflammation. Increased hs-CRP concentrations have been reported in many diseases, including infectious diseases, cardiovascular diseases, diabetes, inflammatory bowel diseases, autoimmune disorders, arthritis, and many cancers.^1^

Recent studies have suggested that hs-CRP level is positively associated with cancer. Two hypotheses have been proposed to explain the relationship between hs-CRP level and cancer.^2^ First, it has been suggested that elevated hs-CRP levels are a result of an underlying cancer. Alternatively, chronic inflammation and elevated hs-CRP might have a causal role in carcinogenesis. In this latter view, inflammation-associated oxidative damage could initiate carcinogenesis by causing inactivating mutations in tumor-suppressor genes or post-translational modifications in proteins involved in DNA repair or apoptotic control. In addition, inflammatory cytokine signaling via intracellular enzymes and transcription factors may inhibit apoptosis and promote the growth and proliferation of cancer cells. Moreover, activation of inflammatory pathways might facilitate tumor progression by promoting cell motility, vascular permeability, and angiogenesis.^3^^,^^4^

To date, epidemiologic evidence of a diagnostic or etiological role of hs-CRP in cancer has been inconsistent. Several epidemiologic studies have attempted to identify associations between baseline hs-CRP and the incidence of human carcinomas, and some have shown positive associations.^5^^–^^8^ The association between hs-CRP and cancer may be site-specific. In a recent meta-analysis of 12 prospective studies,^3^ elevated hs-CRP was associated with an increased risk of incident cancer of any type, lung cancer, and, possibly, colorectal, breast, and ovarian cancers, but not prostate cancer. A few studies in Asian populations have shown positive correlations between plasma CRP and gastric cancer.^8^ To our knowledge, no study has reported specifically on the association between hs-CRP and site-specific cancer risk in Asian populations. In addition, we attempted to examine the association of hs-CRP with pathologic type, because a certain pathologic type, such as adenocarcinoma, is responsible for cancers associated with obesity and diabetes.^9^^–^^11^

Although significant ethnic/racial differences in serum hs-CRP have been observed,^12^ only a few studies have been conducted in Asian populations and fewer still have focused on Koreans. This is the first large-scale study to examine associations between hs-CRP and cancer in Koreans. The aim of the present study was to determine whether hs-CRP is associated with cancer risk or with specific pathologic types or sites of cancer in Koreans. In the site-specific analysis, we focused on common cancers, ie, cancers of the stomach, colon, prostate, and thyroid.

## METHODS

We collected data on 113 403 subjects who underwent medical health check-ups from January 2005 through December 2007 at Asan Medical Center, one of the largest general hospitals in Seoul, South Korea. We used data from the first health check-ups of participants (*n* = 81 779) who had been examined more than once during a 3-year period. Subjects with a history of any cancer and those with concomitant diseases that might raise serum hs-CRP^13^ (eg, infectious diseases, autoimmune conditions, asthma, and osteoarthritis) were excluded (*n* = 998). Cases were defined as individuals with no past history of cancer who received a diagnosis of cancer at the initial examination. Cancer cases were identified by using the medical and pathological records of the same hospital, which is an active and highly regarded cancer treatment center in Korea. Extensive record linkage to ascertain any missed cancer cases, however, was not possible. Individuals who received a cancer diagnosis at their second or third health check-up during the follow-up period were included in the subgroup of incident cancer cases.

All participants gave written informed consent and the institutional review board of Asan Medical Center approved this study. Before the health check-ups, all subjects were asked to complete a medical questionnaire that asked about current symptoms, medical history, present medications, smoking status, alcohol consumption, exercise, and family history of cancer.

All blood pressure and anthropometric measurements were performed by a well-trained medical technician, according to standard techniques. Blood was collected from the antecubital vein of each subject into Vacutainer tubes after an 8-hour fasting period. Plasma glucose, high-density lipoprotein (HDL)-cholesterol, and triglycerides were measured using an autoanalyzer (TBA-200FR, Toshiba, Tokyo, Japan). Serum hs-CRP levels were quantitatively determined by enzyme immunoassay using the automated immunoturbidimetric method (Cobas Integra 800, Roche diagnostics, Basel, Switzerland).

A blood pressure of 140/90 mm Hg or higher was defined as hypertension, and a fasting serum glucose concentration of 126 mg/dL or higher was defined as diabetes mellitus. Individuals using antidiabetic or antihypertensive medications were also considered to have met the criteria for diabetes and hypertension, respectively. Hypertriglyceridemia was defined as a fasting serum triglyceride concentration of 150 mg/dL or higher or treatment with an antihypertriglyceridemic medication. Low HDL was defined as a level lower than 40 mg/dL in men and lower than 50 mg/dL in women.

Body mass index (BMI) was calculated as the weight in kilograms divided by the square of the height in meters. BMI values were categorized based on the Asia-Pacific consensus^14^ as obese (≥25 kg/m^2^), overweight (23–24.9 kg/m^2^), and normal weight or underweight (<23 kg/m^2^). Metabolic syndrome was defined based on the US National Cholesterol Education Program Adult Treatment Panel III (NCEP-ATP III) criteria,^15^ with minor modifications. As detailed in the NCEP-ATP III report, participants satisfying at least 3 of the following 5 criteria were considered to have metabolic syndrome: (1) high blood pressure, defined as higher than 130/85 mm Hg or use of antihypertensive medication, (2) impaired fasting glucose, defined as 100 mg/dL or higher or use of antidiabetic medication, (3) hypertriglyceridemia, defined as 150 mg/dL or higher, (4) low HDL, defined as lower than 40 mg/dL in men and lower than 50 mg/dL in women, and (5) abdominal obesity, defined as a waist circumference of 90 cm or higher in men and 80 cm or higher in women, based on the Asia-Pacific consensus.^14^

Subjects were classified on the basis of smoking status as a current smoker (<20, 20–40, >40 cigarettes/day), ex-smoker, or nonsmoker (never smoker). Consumption of alcohol was also determined, and subjects were grouped by frequency of consumption (≤1, 2 to 3, or ≥4 times per week). Exercise level for each subject was assessed and categorized as none, ≤2, or ≥3 sessions per week. The validity of the alcohol and exercise questions has not been fully established.

Selected characteristics were compared between cases and non-cases using the independent-sample *t* test for numeric variables and the chi-square test for categorical variables. The Wilcoxon rank-sum test was used to evaluate differences in hs-CRP level by case/non-case status because serum hs-CRP is not normally distributed on raw or log-transformed scales. Participants were categorized by hs-CRP level as lower than 1 mg/L, 1 to 3 mg/L, and higher than 3 mg/L, which are the cutoff points proposed in the American Heart Association’s clinical guidelines for cardiovascular disease. These categories approximate tertiles of serum hs-CRP distribution among more than 40 000 adults in more than 15 populations and allow for adequate definition of the distribution.^13^

Odds ratios (ORs) and 95% confidence intervals (95% CIs) for the association of hs-CRP level with cancer risk were estimated using logistic regression analysis. We first estimated the crude OR according to category of hs-CRP and then adjusted for age (20–39, 40–49, 50–59, 60–69, and ≥70 years) and sex (model I). We further performed multivariate analysis to additionally adjust for potential confounding risk factors, which were identified by consulting the relevant literature. These risk factors included BMI (<25, ≥25 kg/m^2^), abdominal obesity (men, ≥90 or <90 cm; women, ≥80 or <80 cm), diabetes, hypertension, dyslipidemia, aspirin use, smoking, alcohol consumption, and exercise (model II). Model III was additionally adjusted for BMI, abdominal obesity, diabetes, hypertension, dyslipidemia, aspirin use, smoking, alcohol consumption, exercise, education level, and monthly income. During this analysis, we dichotomized exercise into active (≥3 sessions/week) versus inactive, drinker (≥4 drinks/week) versus nondrinker, education level (≥12 vs <12 years), and monthly income (≥2500 vs <2500 US dollars).

Analyses evaluated all-cancer risk and the risks for some major primary sites and pathologic types. Likelihood ratio tests were used to assess linear trends in ORs with respect to hs-CRP tertile, which yielded quantitative scores for all levels (1, 2, and 3). We also estimated the associations between hs-CRP and the risk of cancer after excluding cancers detected within 1 year of health examination. All statistical tests and corresponding *P* values were 2-sided, and a *P* value less than 0.05 was considered statistically significant. We analyzed data using the statistical software package SAS version 9.1 (SAS Institute, Cary, NC, USA).

## RESULTS

The demographic characteristics of the study participants are presented in Table 1. A total of 80 052 non-cases, 729 cancer cases with 88 subgroup cancer annotation were included. The mean age of cases and non-cases was 55.1 and 47.6 years, respectively. As compared with non-cases, cases were older (*P* < 0.01), more physically active (*P* < 0.01), less educated (*P* < 0.01), and more likely to be aspirin users (*P* = 0.0002). Cases were also more likely to have comorbid conditions (hypertension, diabetes, or dyslipidemia). Cases and non-cases had similar distributions of sex, smoking status, alcohol consumption, and income.

**Table 1.** Demographic characteristics of study subjects

	Non-cases (*n* = 80 052) No. (%)	Cancer cases (*n* = 729) No. (%)	Incident cancer cases subgroup^a^ (*n* = 88) No. (%)
Mean age^b^ (years)	47.6 ± 10.7	55.1 ± 10.4	56.3 ± 9.8
Sex			
Male	45 485 (56.8)	421 (57.7)	54 (61.4)
Female	34 567 (43.2)	308 (42.3)	34 (38.6)
Smoking status			
Nonsmokers	40 969 (51.2)	371 (51.0)	42 (47.7)
Ex-smokers	19 778 (24.7)	186 (25.5)	25 (28.4)
Current smokers ​ (cigarettes/day)	19 427 (24.1)	171 (23.5)	21 (23.9)
<20	14 614 (18.3)	129 (17.7)	16 (18.2)
20–40	3451 (4.3)	31 (4.2)	5 (4.6)
>40	1240 (1.5)	12 (1.6)	1 (1.1)
Alcohol consumption (times/week)			
≤1	48 766 (60.9)	457 (62.6)	53 (60.2)
2–3	20 558 (25.7)	155 (21.3)	23 (26.1)
≥4	10 727 (13.4)	117 (16.1)	12 (13.7)
Exercise^b^ (sessions/week)			
None	24 007 (30.3)	199 (27.3)	18 (20.5)
1–2	23 380 (29.2)	184 (25.1)	22 (25.0)
≥3	32 663 (40.5)	346 (47.6)	48 (54.5)
Educational level^b^ (years)			
≤12	32 384 (42.1)	384 (57.0)	35 (42.2)
>12	44 560 (57.9)	290 (43.0)	48 (57.8)
Income (US dollars/month)			
≤2500	19 657 (30.2)	206 (36.0)	15 (21.1)
<2500 and ≤4200	17 532 (26.9)	136 (23.7)	17 (23.9)
>4200 and ≤5800	13 070 (20.1)	105 (18.3)	16 (22.6)
>5800	14 840 (22.8)	126 (22.0)	23 (32.4)
Hypertension^b^			
Yes	17 453 (21.8)	262 (36.0)	28 (31.8)
No	62 599 (78.2)	467 (64.0)	60 (68.2)
Diabetes^b^			
Yes	5613 (7.0)	116 (15.9)	14 (15.9)
No	74 439 (93.0)	613 (84.1)	74 (84.1)
Dyslipidemia^b^			
Yes	3310 (4.1)	46 (6.3)	3 (3.4)
No	76 742 (95.9)	683 (93.7)	85 (96.6)
Aspirin use^b^			
Yes	3993 (5.0)	59 (8.1)	10 (11.4)
No	76 059 (95.0)	670 (91.9)	78 (88.6)

^a^Cancer cases detected later than 1 year after health examination.

^b^*P* < 0.05 between non-cases and cases by Student *t*-test or chi-square test.

The anthropometric and laboratory characteristics of the study participants are shown in Table 2. As compared with non-cases, cases had a higher BMI (*P* = 0.047), larger waist circumference (*P* < 0.01), higher blood pressure (*P* < 0.01), higher serum fasting glucose (*P* < 0.01), lower HDL cholesterol (*P* < 0.01), and a higher prevalence of metabolic syndrome (*P* < 0.01). Serum triglyceride levels were not significantly different between cases and non-cases. Mean serum hs-CRP level was significantly higher among cases (2.9 mg/L) than non-cases (1.4 mg/L; *P* < 0.01, Wilcoxon rank-sum test). The demographic, anthropometric, and laboratory characteristics of the subgroup cases were not significantly different from those of the cancer cases.

**Table 2.** Anthropometric and laboratory characteristics of study subjects

	Non-cases (*n* = 80 052) Mean ± SD	Cancer cases (*n* = 729) Mean ± SD	Incident cancer cases subgroup^a^ (*n* = 88) Mean ± SD	hs-CRP (mg/L) Mean ± SD
BMI^b^ (kg/m^2^)	23.7 ± 3.0	24.0 ± 3.0	23.8 ± 2.7	
<23^c^	32 832 (41.1)	276 (37.7)	35 (39.8)	1.2 ± 4.3
≥23 and <25^c^	21 104 (26.4)	202 (27.8)	27 (30.7)	1.4 ± 3.6
≥25^c^	26 039 (32.5)	251 (34.5)	26 (29.5)	1.8 ± 4.2
Waist circumference^b^ (cm)	81.0 ± 9.2	82.3 ± 9.3	82.0 ± 8.6	
Blood pressure (mm Hg)				
Systolic^b^	119.7 ± 15.7	124.6 ± 16.7	122.4 ± 16.0	
Diastolic^b^	74.6 ± 9.6	76.4 ± 9.2	75.4 ± 8.8	
Fasting blood glucose^b^ (mg/dL)	97.8 ± 20.2	103.5 ± 23.8	101.4 ± 23.2	
Triglyceride (mg/dL)	128.8 ± 86.1	130.4 ± 78.5	129.2 ± 82.0	
HDL-cholesterol^b^ (mg/dL)	55.6 ± 14.0	53.8 ± 14.3	54.3 ± 13.5	
Metabolic syndrome^b^				
Yes^c^	15 314 (19.1)	193 (26.5)	18 (20.5)	1.9 ± 4.5
No^c^	64 738 (80.9)	536 (73.5)	70 (79.5)	1.3 ± 4.0
hs-CRP(mg/L)^b^				
Mean ± SD	1.4 ± 4.0	2.9 ± 9.3	1.6 ± 1.5	
Median (IQR)	0.7 (0.4–1.2)	0.8 (0.5–1.9)	0.9 (0.6–1.9)	
<1	52 922 (66.1)	398 (54.6)	45 (51.1)	
1–3	20 870 (26.1)	214 (29.4)	33 (37.5)	
>3	6260 (7.8)	117 (16.0)	10 (11.4)	

BMI: body mass index; HDL: high-density lipoprotein; hs-CRP: high-sensitivity C-reactive protein; SD: standard deviation; IQR: interquartile range.

^a^Cancer cases detected later than 1 year after health examination.

^b^*P* < 0.05 between controls and cases by Student *t*-test, chi-square test, or Wilcoxon test.

^c^No. (%).

Mean serum hs-CRP level was significantly higher in subjects with metabolic syndrome (1.9 mg/L) than in those without metabolic syndrome (1.3 mg/L; *P* < 0.01, Wilcoxon rank-sum test). We also analyzed the association between hs-CRP and cancer according to the metabolic syndrome. The results are shown in Table 3. After stratification, the positive associations persisted in subjects without metabolic syndrome. The adjusted ORs (model III) for cancer in subjects with metabolic syndrome were 1.51 (95% CI = 1.05–2.17) in the second highest hs-CRP category and 2.19 (95% CI =1.38–3.45) in the highest hs-CRP category, as compared with the lowest category (*P* for trend = 0.0006). The adjusted ORs (model III) for cancer in subjects without metabolic syndrome were 1.04 (95% CI = 0.81–1.34) in the second highest hs-CRP category and 1.92 (95% CI = 1.41–2.63) in the highest hs-CRP category, as compared with the lowest category (*P* for trend = 0.0009).

**Table 3.** Odds ratios (ORs) and 95% confidence intervals for cancer by serum hs-CRP category and metabolic syndrome status

	Category of hs-CRP (mg/L)			*P* for trend
	
	<1	1–3	>3	
With metabolic syndrome				
Cases, *n*	77	81	36	
Crude OR	1	1.28 (0.94–1.75)	1.93 (1.30–2.88)	0.0017
Model I^a^	1	1.28 (0.93–1.75)	1.86 (1.24–2.77)	0.0030
Model II^b^	1	1.27 (0.92–1.74)	1.82 (1.21–2.72)	0.0045
Model III^c^	1	1.51 (1.05–2.17)	2.19 (1.38–3.45)	0.0006
Without metabolic syndrome				
Cases, *n*	321	133	81	
Crude OR	1	1.28 (1.04–1.56)	2.57 (2.01–3.29)	<0.0001
Model I^a^	1	1.13 (0.92–1.39)	2.15 (1.68–2.77)	<0.0001
Model II^b^	1	1.10 (0.89–1.37)	2.05 (1.57–2.68)	<0.0001
Model III^c^	1	1.04 (0.81–1.34)	1.92 (1.41–2.63)	0.0009

^a^Adjusted for age and sex.

^b^Additionally adjusted for BMI, diabetes, hypertension, dyslipidemia, smoking, alcohol consumption, exercise, and aspirin use.

^c^Additionally adjusted for BMI, diabetes, hypertension, dyslipidemia, smoking, alcohol consumption, exercise, aspirin use, education level, and income.

Overall, the prevalence ORs for cancer were positively associated with increasing categories of hs-CRP (<1, 1–3, >3 mg/L). Crude and adjusted ORs for cancer are presented in Table 4. The crude ORs for cancer were 1.36 (95% CI = 1.16–1.62) for the second highest hs-CRP category and 2.49 (95% CI = 2.02–3.07) for the highest hs-CRP category, as compared with the lowest hs-CRP category (*P* for trend <0.0001). After adjustment for age, sex, BMI, abdominal obesity, diabetes, hypertension, dyslipidemia, aspirin use, smoking, alcohol consumption, exercise, education level, and income, the positive associations were weaker, but remained. The adjusted ORs for cancer were 1.16 (95% CI = 0.95–1.42) for the second highest hs-CRP category and 1.94 (95% CI = 1.50–2.50) for the highest hs-CRP category, as compared with the lowest hs-CRP category (*P* for trend <0.0001). The association was not attenuated after excluding cancer cases detected within 1 year of the health examination. These results are presented in Table 4.

**Table 4.** Odds ratios (ORs) and 95% confidence intervals for cancer by serum hs-CRP category

	Category of hs-CRP (mg/L)			*P* for trend
	
	<1	1–3	>3	
Cases, *n*	397	214	117	
Crude OR	1	1.36 (1.16–1.62)	2.49 (2.02–3.07)	<0.0001
Model I^a^	1	1.18 (1.00–1.40)	2.04 (1.65–2.52)	<0.0001
Model II^b^	1	1.15 (0.96–1.37)	1.93 (1.55–2.42)	<0.0001
Model III^c^	1	1.16 (0.95–1.42)	1.94 (1.51–2.51)	<0.0001

Excluding cancer cases detected within 1 year of health examination				

Cases, *n*	45	33	10	
Crude OR	1	1.86 (1.19–2.92)	2.35 (1.18–4.66)	0.0055
Model I^a^	1	1.54 (0.98–2.43)	1.83 (0.92–3.66)	0.0806
Model II^b^	1	1.38 (0.82–2.30)	2.04 (0.98–4.25)	0.0446
Model III^c^	1	1.56 (0.88–2.75)	2.28 (1.00–5.25)	0.0280

^a^Adjusted for age and sex.

^b^Additionally adjusted for BMI, diabetes, hypertension, dyslipidemia, smoking, alcohol consumption, exercise, and aspirin use.

^c^Additionally adjusted for BMI, diabetes, hypertension, dyslipidemia, smoking, alcohol consumption, exercise, aspirin use, education level, and income.

Subjects were stratified according to sex to assess the sex-specific association of hs-CRP and cancer. After stratification, as compared with the lowest hs-CRP category, the positive associations were stronger for women than for men in the second highest hs-CRP category and for men as compared with women in the highest hs-CRP category. The adjusted ORs (model III) for cancer in men were 1.15 (95% CI =0.90–1.48) in the second highest hs-CRP category and 2.15 (95% CI = 1.60–2.90) in the highest hs-CRP category, as compared with the lowest category (*P* for trend <0.0001). The adjusted ORs (model III) for cancer in women were 1.23 (95% CI = 0.87–1.75) in the second highest hs-CRP category and 1.43 (95% CI = 0.85–2.41) in the highest hs-CRP category, as compared with the lowest category (*P* for trend = 0.11).

Next, we performed separate analyses to determine whether hs-CRP was associated with specific pathologic types of cancer. The results are shown in Table 5. Among a total of 729 cancer cases, 607 (83.4%) were adenocarcinomas, 18 (2.5%) were squamous cell carcinomas, and 104 (14.1%) were another histologic type. Adenocarcinoma was positively associated with hs-CRP. The adjusted ORs (model III) for adenocarcinoma were 1.15 (95% CI = 0.92–1.43) for the second highest hs-CRP category and 2.04 (95% CI = 1.56–2.68) for the highest hs-CRP category, as compared with the lowest hs-CRP category (*P* for trend <0.0001). There was no significant association between hs-CRP and squamous cell carcinoma.

**Table 5.** Odds ratios (ORs) and 95% confidence intervals for cancer histology and type by serum hs-CRP category

	Category of hs-CRP (mg/L)			*P* for trend
	
	<1	1–3	>3	
Cancer histology				
Adenocarcinoma				
Cases, *n*	328	177	102	
Crude OR	1	1.37 (1.14–1.65)	2.64 (2.11–3.30)	<0.0001
Model I^a^	1	1.18 (0.98–1.42)	2.13 (1.70–2.67)	<0.0001
Model II^b^	1	1.16 (0.95–1.41)	2.06 (1.62–2.62)	<0.0001
Model III^c^	1	1.15 (0.92–1.43)	2.04 (1.56–2.68)	<0.0001
Squamous cell carcinoma				
Cases, *n*	8	8	2	
Crude OR	1	2.54 (0.95–6.76)	2.11 (0.45–9.96)	0.100
Model I^a^	1	2.23 (0.83–6.01)	1.75 (0.37–8.36)	0.1981
Model II^b^	1	1.45 (0.48–4.34)	0.75 (0.09–6.15)	0.9262
Model III^c^		—	—	
Others				
Cases, *n*	62	29	13	
Crude OR	1	1.19 (0.76–1.84)	1.77 (0.97–3.23)	0.0729
Model I^a^	1	1.08 (0.69–1.69)	1.57 (0.86–2.86)	0.2024
Model II^b^	1	1.06 (0.67–1.69)	1.44 (0.77–2.72)	0.3702
Model III^c^	1	1.19 (0.69–2.05)	1.67 (0.80–3.48)	0.2158

Cancer type				
Stomach cancer				
Cases, *n*	99	53	28	
Crude OR	1	1.36 (0.97–1.90)	2.39 (1.57–3.64)	<0.0001
Model I^a^	1	1.10 (0.78–1.54)	1.79 (1.17–2.74)	0.0201
Model II^b^	1	1.14 (0.81–1.62)	1.75 (1.12–2.73)	0.0165
Model III^c^	1	1.11 (0.75–1.64)	1.66 (1.00–2.77)	0.0622
Colon cancer				
Cases, *n*	93	38	27	
Crude OR	1	1.04 (0.71–1.51)	2.45 (1.60–3.77)	0.0011
Model I^a^	1	0.91 (0.62–1.33)	2.07 (1.34–3.19)	0.0191
Model II^b^	1	0.91 (0.61–1.35)	2.06 (1.31–3.25)	0.0287
Model III^c^	1	0.97 (0.63–1.49)	2.21 (1.35–3.61)	0.0165
Prostate cancer				
Cases, *n*	27	16	10	
Crude OR	1	1.50 (0.81–2.79)	3.13 (1.52–6.47)	0.0031
Model I^a^	1	1.01 (0.54–1.87)	1.64 (0.79–3.42)	0.2803
Model II^b^	1	0.97 (0.50–1.89)	1.88 (0.89–3.99)	0.1162
Model III^c^	1	0.82 (0.37–1.85)	1.83 (0.75–4.43)	0.2844
Thyroid cancer				
Cases, *n*	98	52	13	
Crude OR	1	1.35 (0.96–1.88)	1.12 (0.63–2.00)	0.2156
Model I^a^	1	1.44 (1.02–2.03)	1.19 (0.66–2.13)	0.1202
Model II^b^	1	1.30 (0.90–1.87)	0.98 (0.52–1.85)	0.7859
Model III^c^	1	1.24 (0.81–1.88)	0.80 (0.36–1.76)	0.8198

^a^Adjusted for age and sex.

^b^Additionally adjusted for BMI, diabetes, hypertension, dyslipidemia, smoking, alcohol consumption, exercise, and aspirin use.

^c^Additionally adjusted for BMI, diabetes, hypertension, dyslipidemia, smoking, alcohol consumption, exercise, aspirin use, education level, and income.

To determine whether hs-CRP was associated with cancer site, we performed analyses stratified by major primary cancer site. The results are shown in Table 5. Cancer sites for which there were fewer than 50 cases were not examined owing to limited statistical power. Of the 729 cancer cases, 180 (24.6%) were stomach cancer, 163 (22.4%) were thyroid cancer, 158 (21.7%) were colorectal cancer, and 53 (7.3%) were prostate cancer. These 4 common cancers collectively accounted for 76% of all cancer cases. The analysis by primary cancer site yielded imprecise estimates, probably due to the limited number of cases; however, several trends were evident. Stomach cancer was marginally positively associated with hs-CRP. The adjusted OR (model III) for stomach cancer was 1.66 (95% CI = 1.00–2.77) for the highest hs-CRP category as compared with the lowest hs-CRP category (*P* for trend =0.0622). Colorectal cancer was positively associated with serum hs-CRP level. The adjusted OR (model III) for colorectal cancer was 2.21 (95% CI = 1.35–3.61) for the highest hs-CRP category as compared with the lowest hs-CRP category (*P* for trend = 0.0165). Prostate cancer was not associated with elevated serum hs-CRP level. The adjusted OR (model III) for prostate cancer was 1.83 (95% CI = 0.75–4.43) for the highest hs-CRP category as compared with the lowest hs-CRP category (*P* for trend = 0.2844). There was no significant association between serum hs-CRP level and thyroid cancer.

## DISCUSSION

We investigated the association between hs-CRP and cancer risk and found that serum hs-CRP was positively associated with cancer. This association was not diminished after excluding cancer cases detected within 1 year of health examination. These findings suggest that elevated hs-CRP may play a role in the pathogenesis of cancer. However, these results do not exclude the possibility that hs-CRP elevation is the consequence of undetected, long-latency cancers.

A preponderance of studies reported that hs-CRP is much higher in cancer patients than in healthy subjects.^16^^–^^18^ Elevated hs-CRP is associated with progressive disease and decreased survival in patients with several cancers, including esophageal, gastric, colorectal, liver, pancreatic, urinary bladder, kidney, ovarian, and cervical cancers.^19^^–^^23^ A number of epidemiologic studies have examined the association between hs-CRP and cancer risk, and some prospective studies have shown a higher risk of developing cancer in people with elevated hs-CRP.^24^^–^^28^ However, not all published studies have confirmed an association between elevated hs-CRP and a higher risk for cancer.^29^^–^^31^

In a recent meta-analysis of 12 prospective studies,^3^ elevated hs-CRP was associated with an increased risk of any cancer (random-effects estimate [RE] = 1.10, 95% CI =1.02–1.18) and lung cancer (RE = 1.32, 95% CI = 1.08–1.61). The associations with hs-CRP were weaker for colorectal (RE = 1.09, 95% CI = 0.98–1.21), breast (RE = 1.10, 95% CI = 0.97–1.26), and ovarian (RE = 1.14, 95% CI = 0.99–1.32) cancer. Serum hs-CRP appeared to be unrelated to prostate cancer risk (RE = 1.00, 95% CI = 0.88–1.13).

In another recent, large, prospective study,^27^ a baseline hs-CRP level higher than 3, versus one lower than 1 mg/L, was associated with a multivariate-adjusted hazard ratio of 1.3 (95% CI, 1.0–1.6) for cancer of any type, 2.2 (95% CI, 1.0–4.6) for lung cancer, 1.9 (95% CI, 0.8–4.6) for colorectal cancer, and 0.7 (95% CI, 0.4–1.4) for breast cancer. The multivariate-adjusted hazard ratio for early death in patients with cancer was 1.8 (95% CI, 1.2–2.7) for an hs-CRP level higher than 3 versus one lower than 1 mg/L.

Despite the considerable heterogeneity of the results from meta-analyses, our findings lend support to the hypothesis that hs-CRP is positively associated with all-cancer risk. However, estimates of cancer site-specific associations with hs-CRP differed. Colorectal and stomach cancer were positively associated with hs-CRP, a result that is consistent with those of a previous meta-analysis^3^ and the Japan Public Health Center-based prospective study.^8^ However, we observed no association between prostate cancer and hs-CRP, a result that corresponds with prospective data from the Rotterdam Study.^25^

We assessed sex-specific associations between hs-CRP and cancer, because several studies have indicated that such differences exist.^32^ After sex stratification, we found that hs-CRP was positively associated with cancer. Our findings differ from those of Zhang et al, who observed no clear relationship between hs-CRP and colorectal cancer risk among subjects enrolled in the Women’s Health Study.^33^

In this study, cancer cases had a higher prevalence of metabolic syndrome than did non-cases. To exclude the possibility of confounding from metabolic syndrome, we conducted stratified analysis by metabolic syndrome status to assess the associations between hs-CRP and cancer. After stratification, positive associations persisted in subjects without metabolic syndrome, which indicates that high hs-CRP is an independent risk factor for cancer.

Our findings regarding histology- and site-specific associations suggest that hs-CRP is more strongly associated with adenocarcinoma. This is compatible with previous findings, which indicate that pan-adenocarcinoma is associated with obesity^9^^–^^11^ and that hs-CRP is higher in obese adults and those with metabolic syndrome.

The present study has several strengths. The large sample size permitted us to evaluate associations of hs-CRP with all cancers and cancers of specific sites and pathologic types. In addition, we adjusted for several important potential confounders, including age, sex, BMI, diabetes, hypertension, dyslipidemia, smoking, alcohol consumption, exercise, aspirin use, education level, and income. To our knowledge, this is the first study to examine the associations between hs-CRP and cancer in Koreans.

There were some limitations, however. First, the study subjects may not have been representative of the general population. The participants in this study were recruited from among adults who visited our hospital for regular health check-ups; thus, they were relatively health conscious. Selection bias may have led to an underestimation of the true excess risk. Second, our study was cross-sectional. Therefore, causality cannot be inferred in the association of hs-CRP with cancer risk. Third, detailed information on lifestyle was not available in the data used in this study. We cannot exclude the possibility that the self-reported questionnaires provided only a rough assessment of alcohol consumption and exercise status. We also recognize that a single measurement of hs-CRP may not represent an individual’s inflammatory status during the development of cancer and that measured levels may be influenced by diurnal or stress-induced variation. However, misclassification bias is unlikely because stress-induced activation of hs-CRP should not have differed by case/non-case status. Finally, the cancer site-specific analyses were based on a small numbers of cancer cases, which highlights the need for further large prospective studies.

In conclusion, we have shown a positive association between serum hs-CRP level and cancer risk. This finding supports the hypothesis that chronic low-grade systemic inflammation increases the risk for cancer. Large prospective studies are necessary to determine the role of hs-CRP in the etiology of cancer.
